# Analysis of inflammatory markers and tau deposits in an autopsy series of nine patients with anti-IgLON5 disease

**DOI:** 10.1007/s00401-023-02625-6

**Published:** 2023-08-30

**Authors:** Evelyn Berger-Sieczkowski, Verena Endmayr, Carmen Haider, Gerda Ricken, Philipp Jauk, Stefan Macher, Walter Pirker, Birgit Högl, Anna Heidbreder, Peter Schnider, Eszter Bradley-Zechmeister, Sara Mariotto, Inga Koneczny, Raphael Reinecke, Gregor Kasprian, Corinna Weber, Melanie Bergmann, Ivan Milenkovic, Thomas Berger, Carles Gaig, Lidia Sabater, Francesc Graus, Ellen Gelpi, Romana Höftberger

**Affiliations:** 1 Department of Neurology, Medical University of Vienna, Vienna, Austria; 2 Comprehensive Center for Clinical Neurosciences and Mental Health, Medical University of Vienna, Waehringer Guertel 18-20, 1090 Vienna, Austria; 3 Division of Neuropathology and Neurochemistry, Department of Neurology, Medical University of Vienna, Waehringer Guertel 18-20, 1090 Vienna, Austria; 4 Center for Medical Physics and Biomedical Engineering, Medical University of Vienna, Vienna, Austria; 5 Department of Neurology, Klinik Ottakring, Vienna, Austria; 6 Department of Neurology, Medical University of Innsbruck, Innsbruck, Austria; 7 Department of Neurology, Landesklinikum Wiener Neustadt, Wiener Neustadt, Austria; 8 Neurology Unit, Department of Neurosciences, Biomedicine, and Movement Sciences, University of Verona, Verona, Italy; 9 Division of Neuroradiology and Musculoskeletal Radiology, Department of Biomedical Imaging and Image-Guided Therapy, Medical University of Vienna, Vienna, Austria; 10 Neurology Service, Hospital Clínic of Barcelona, Barcelona, Spain; 11 Neuroimmunology Laboratory-Institut d’Investigacions Biomèdiques August Pi i Sunyer (IDIBAPS), Barcelona, Spain; 12 Neurological Tissue Bank of the Biobanc-Hospital Clinic-IDIBAPS, Barcelona, Spain

## Abstract

Anti-IgLON5 disease is a rare neurological, probably autoimmune, disorder associated in many cases with a specific tauopathy. Only a few post-mortem neuropathological studies have been reported so far. Little is known about the pathogenic mechanisms that result in neurodegeneration. We investigated the neuropathology of anti-IgLON5 disease and characterized cellular and humoral inflammation. We included nine cases (six of them previously published). Median age of patients was 71 years (53–82 years), the median disease duration was 6 years (0.5–13 years), and the female to male ratio was 5:4. Six cases with a median disease duration of 9 years presented a prominent tauopathy. Five of them had a classical anti-IgLON5-related brainstem tauopathy and another presented a prominent neuronal and glial 4-repeat tauopathy, consistent with progressive supranuclear palsy (PSP). Three cases with short disease duration (median 1.25 years) only showed a primary age-related neurofibrillary pathology. Inflammatory infiltrates of T and B cells were mild to moderate and did not significantly differ between anti-IgLON5 disease cases with or without tauopathy. In contrast, we found an extensive neuropil deposition of IgG4 in the tegmentum of the brainstem, olivary nucleus, and cerebellar cortex that was most prominent in two patients with short disease duration without the typical IgLON5-related tauopathy. The IgG4 deposits were particularly prominent in the cerebellar cortex and in these regions accompanied by mild IgG1 deposits. Activated complement deposition (C9neo) was absent. Our study indicates that IgLON5-related tau pathology occurs in later disease stages and may also present a PSP-phenotype with exclusively 4-repeat neuronal and glial tau pathology. The prominent deposition of anti-IgLON5 IgG4 at predilection sites for tau pathology suggests that anti-IgLON5 antibodies precede the tau pathology. Early start of immunotherapy might prevent irreversible neuronal damage and progression of the disease, at least in a subgroup of patients.

**Supplementary Information:**

The online version contains supplementary material available at 10.1007/s00401-023-02625-6.

## Introduction

Anti-IgLON5 disease is a neurological disorder that is associated with autoantibodies against IgLON5, a cell adhesion molecule belonging to the IgLON (immunoglobulin LAMP, OBCAM and neurotrimin) subgroup of the immunoglobulin superfamily and strongly expressed in neurons [24]. The clinical manifestations are variable and commonly present as a sleep disorder, bulbar syndrome, cognitive impairment, neuromuscular manifestations, or movement disorders [7]. The movement disorders can be prominent and in 14% of anti-IgLON5 disease patients, initial symptoms may suggest a diagnosis of progressive supranuclear palsy (PSP) [3, 6, 7]. The disease shows a strong association with the human leukocyte antigen (HLA) haplotype DRB1*10:01 and DQB1*05:01. IgLON5 IgG are composed of a variable ratio of IgG1 and IgG4 subclasses with a predominance of IgG4 antibodies in the majority of patients [8]. IgLON5 IgG1 antibodies irreversibly internalize IgLON5, which results in disruption of the cytoskeletal organization in cultured rat hippocampal neurons [17]. Moreover, additional experiments in transfected human embryonic kidney (HEK) cells and hippocampal neurons revealed that patients´ IgLON5 antibodies disrupt the interaction of secreted IgLON5 ectodomain with other members of the IgLON family [18]. Initial neuropathological studies in anti-IgLON5 disease showed a neuronal tauopathy, mainly involving the hypothalamus and tegmentum of the brainstem and suggested a primary neurodegenerative disease [11, 24]. However, a few patients with severe anti-IgLON5 disease did not show a tauopathy at autopsy, suggesting that immunological mechanisms may precede neurodegeneration [4, 5].

In the current study, we investigated post-mortem brain tissue of nine anti-IgLON5 patients to characterize the cellular and humoral inflammation and the spectrum of IgLON5-antibody-associated tauopathy.

## Material and methods

### Patients

We investigated post-mortem brain tissue from nine patients with anti-IgLON5 disease, including three novel and six previously published cases. Among the latter were five with confirmed IgLON5 antibodies and one with probable IgLON5 disease [4, 5, 11, 14, 22]. IgLON5 antibodies were identified with standardized methods comprising an in-house tissue-based and cell-based assay as previously published [24]. For comparison, we additionally examined autopsy brain tissue from six patients with clinically and neuropathologically confirmed PSP, without IgLON5 antibodies in CSF, and four controls. HLA typing was either performed from leukocyte pellets from EDTA blood or frozen post-mortem brain tissue. The study was approved by the Institutional Review Board of the Medical University of Vienna (EK 1123/2015; 1454/18; 1636/2019).

### Neuropathological analysis

Neuropathological investigations were performed on formalin-fixed and paraffin-embedded (FFPE) brain tissue comprising hippocampus, basal ganglia, brainstem, cerebellum, and spinal cord. Five cases were obtained from the archive of the Division of Neuropathology and Neurochemistry, Dept. of Neurology, Medical University of Vienna, two cases from the Barcelona-HC-IDIBAPS Brain Bank, one from Navarra Hospital in Spain [5, 11] and one from the Neurology Unit, University of Verona [4].

As “tauopathy controls” we included six autopsy cases of PSP with negative IgLON5 antibodies in CSF (control A), two systemic disorders (one sepsis, one acute hypoxic brain damage) (control B), one anti-NMDAR encephalitis (positive control for IgG1 deposits), and one non-neurological control. For detailed neuropathological analysis, slides were stained with hematoxylin & eosin and Luxol Fast Blue myelin stain. To assess neurodegeneration-related changes, we stained for Bielschowsky silver impregnation, anti-phosphorylated tau (AT8; Thermo Scientific, Rockford, IL, USA), anti-RD4 tau (Millipore, Temecula, CA, USA), anti-RD3 tau (Millipore, Temecula, CA, USA), anti-betaA4-amyloid (DAKO, Glostrup, Denmark), anti-pTDP-43 (Abnova, Taipei, Taiwan), and anti-alpha-synuclein (Novocastra, Newcastle, UK). To investigate inflammation, we applied anti-CD3 (Leica Biosystems), CD8 (DAKO, Glostrup, Denmark), CD4 (DAKO, Glostrup, Denmark), CD20 (DAKO, Glostrup, Denmark), CD79a (DAKO, Glostrup, Denmark), HLADR (DAKO, Glostrup, Denmark), MHC class I (mouse monoclonal IgG2a kappa antibody HC10; gift from Hidde Ploegh, [26] granzyme B (Neomarkers), IgG (DAKO, Glostrup, Denmark), IgG1 (Sigma-Aldrich, St. Louis, MO, USA), IgG4 (Bio-Rad, Hercules, CA, USA), IgLON5 (rabbit polyclonal IgG antibody targeting amino acids 218–289 of human IgLON5, ab122763; Abcam, Cambridge, UK), PD1 (Abcam, Cambridge, UK), and terminal complement complex C9neo (rabbit polyclonal antibody, RM-9107-S; gift from Professor Paul Morgan, Cardiff, UK) antibodies. Antibody binding was visualized with 3,3´-diaminobenzidine (DAB).

To analyse the co-localization of IgG4 deposits and IgLON5 clusters, FFPE tissue sections were examined by immunofluorescence double labelling: sections were first incubated with a primary antibody against IgG4 (polyclonal mouse, dilution 1:100 in 1xPBS, BIO-RAD MCA2098) for two hours at room temperature, followed by administration of both, anti-IgG4 (1:100 in 1xPBS) and commercial anti-IgLON5 (polyclonal, rabbit, dilution 1:100 in 1xPBS, Abcam ab122763) antibody for 4 °C overnight. For detection of the IgLON5 primary antibody, an AF488-conjugated anti-rabbit secondary antibody (green) was applied. To amplify the IgG4 signal a Catalyzed Signal Amplification (CSA) system (DAKO®, K1500) was used, followed by applying Cy3-conjugated anti-streptavidin secondary antibody (red). Finally, nuclear staining was performed using 4′,6-Diamidino-2-phenylindole-dihydrochloride (DAPI), sections were mounted with Aqua-Poly/Mount (PolySciences, 18,606–20) and visualized with a confocal laser microscope (Zeiss LSM 700, Core Facility Medical University of Vienna).

Quantification of inflammation.

The amount of perivascular and parenchymal CD3, CD4, CD8, CD20, CD79a, and PD1 positive T and B cells was evaluated in anti-IgLON5 disease cases, PSP without IgLON5 antibodies, and three controls (one sepsis, one acute hypoxic brain damage, one non-neurological control) in the tegmentum of the brainstem (pons or medulla oblongata – areas with the highest tau burden in anti-IgLON5 disease) with an ocular morphometric grid, in an area with maximum of inflammation measuring 1 mm^2^. Deposition of IgG1, IgG4, and IgG was evaluated semi-quantitatively (mild: + ; moderate: +  + ; pronounced: +  + +) in available sections of eight anti-IgLON5 disease cases, seronegative PSP, and three controls (one anti-NMDAR-encephalitis, one sepsis, one acute hypoxic brain damage) in the hippocampus (CA1, CA2, CA3, and CA4 subfields), hypothalamus, pons (tegmentum and basis), medulla oblongata (tegmentum, nucleus olivaris inferior), cerebellum (cortex, nucleus dentatus), and spinal cord (anterior horn, posterior horn), see supplementary Table 1 and 2, online resource.

### Flow cytometry analysis of IgG subclasses

HEK293T cells were transfected with DNA encoding IgLON5 or no DNA using polyethyleneimine. 1 × 10^5^ cells were incubated with a 1:10 dilution of patient CSF in a staining medium (DMEM Sigma-Aldrich, D6429) supplemented with 1% penicillin/streptomycin (Sigma-Aldrich, P4333), 1% BSA, 20 mM HEPES and 10% donkey serum) for 20 min at 4 °C. After washing the cells in 500 µl medium, cells were fixed for 10 min in 4% paraformaldehyde, washed, and incubated with a 1:500 dilution of biotinylated mouse anti-human IgG1 (B6775), IgG2 (B3398), IgG3 (B3523) or IgG4 (B3648, all by Sigma Aldrich) in the staining medium for 30 min at 4 °C. After washing, the cells were incubated with Cy3-streptavidin 1:750 in staining medium, 30 min at 4 °C in the dark. Cells were washed and resuspended in 1xPBS supplemented with 2.5 mM EDTA, pH 8.0 before analysis with a Cytoflex LX flow cytometer (Beckman Coulter). Cells with a positive signal for AF488 were gated and analysed for median PE fluorescence intensity, and as a control for unspecific binding, the median PE fluorescence intensity of mock transfected or total cells for every sample was measured and subtracted [16].

## Results

### Patients

We included nine patients with anti-IgLON5 disease (eight definite, one [patient 8] probable [antibody status missing but neuropathological changes compatible with anti-IgLON5 disease as reported [22]]), the female:male ratio was 5:4, the median age was 71 years (range: 53–82 years). The median duration of the disease was 6 years (range: 0.5–13 years). Five patients (55.5%) had the classical HLA haplotype (DQB1*05:01 & DRB1*10:01). Seven cases were diagnosed as anti-IgLON5 disease during their lifetime and two cases were retrospectively identified as anti-IgLON5 disease. Clinical information including HLA subclass and treatments are summarized in Table 1.

**Table 1:** Clinical manifestations, presence of brainstem tau pathology, and HLA subclass in nine patients with anti-IgLON5 disease

Patient [ref]	Age, sex	Disease duration	Clinical presentation	Brainstem tau- pathology	% IgG4 (serum)	HLA type	Immunotherapy
1	67, M	9 years	See text	Yes (PSP)	80%*	DRB1*01:02 DRB1*03:01 DQB1*02:01 DQB1*05:01	No
2 [4]	69, F	15 months	Recurrent episodes of syncope, anxiety, obsessive thinking, compulsions, mild downgaze palsy, parasomnia including continuous limb movements and lip sucking during night time sleep, central apnea, nocturnal hypoxemia and bradycardia, cognitive decline with MMSE 16	No	n.a	DRB1*03:01 DRB1*16:01 DQB1*05:02 DQB1*02:01	Steroids
3 [5]	71, M	2 years	Insomnia with nocturnal confusion and enuresis, mild forgetfulness followed by gait disturbance, ptosis, dysphagia, dysarthria, velopalatine and oromandibular dyskinesias, spontaneous myoclonus, postural tremor of upper limbs	No	75%	DRB1*10:01 DQB1*05:01	IVIG
4 [14]	54, F	13 years	Dysphagia, mild bilateral ptosis, slightly broad-based gait. Insomnia with daytime sleepiness, unintentional sleep episodes at work, parasomnia (talking, hand and leg jerking), apnea-hypopnea index 14.6/h, nocturnal stridor, severe episodes of respiratory insufficiency	Yes	76%	DRB1*10:01 DQB1*05:01	No
5 [24]	53 M	6 years	Sleep disorder including parasomnia, sleep apnea, stridor, mild excessive daytime sleepiness, mild dysphagia and enuresis	Yes	71%	DRB1*10:01 DQB1*05:01	Steroids, cyclophosphamide
6 [24]	76, F	6 months	Severe gait instability, parasomnia, sleep apnea, stridor, saccadic intrusions on pursuit, dysphagia, dysarthria, vocal cord paresis, central hypoventilation	Yes	45%	DRB1*10:01 DQB1*05:01	Steroids, cyclophosphamide
7	82, M	6 months	See text	No	96%	DRB1*10:01 DRB1*15:01 DQB1*05:01 DQB1*06:02	IVIG, rituximab
8 [22]	77, F	10 years	Probable anti-IgLON5 disease. Initial dyspnea and dysphagia, later stridor, hallucinations and confusion. Bilateral vocal cord paralysis, absent tendon reflexes and pan-hypoesthesia distal in both lower extremities; died unexpectedly during sleep	Yes	n.a	n.a	No
9	76, F	9 years	See text	Yes	n.a	DRB1*01 DRB1*11 DQB1*03 DQB1*05	Steroids, IVIG, rituximab

*M* male, *F* female, *n.a*. not available, *IVIG* intravenous immunoglobulins

*IgG4 in CSF (serum not available)

### Clinical history of the three unreported IgLON5 patients

**Fig. 1:**
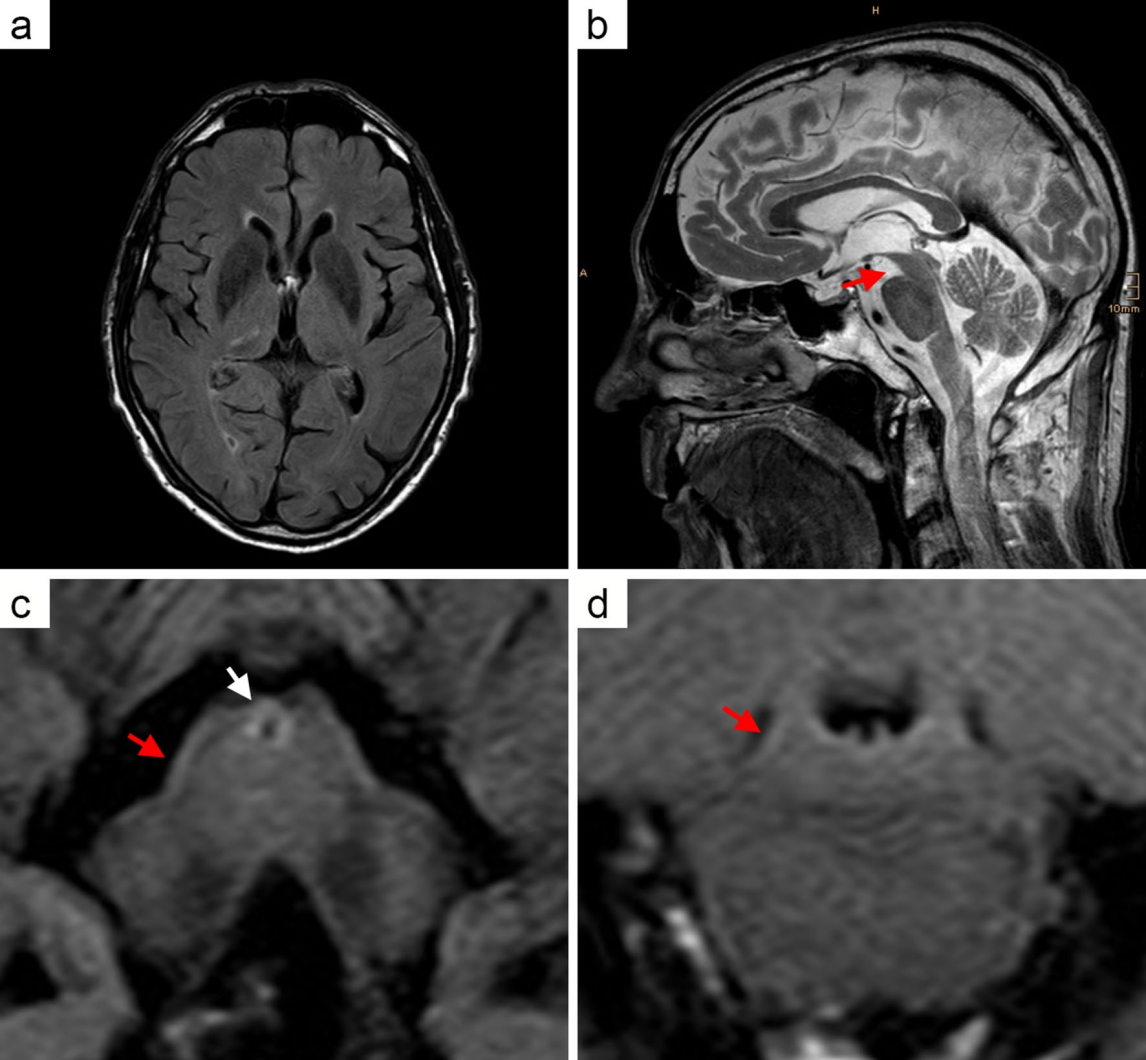
Brain MRI scan of patient 1 with PSP neuropathology. Brain MRI scan (axial, fluid-attenuated recovery sequences (FLAIR)) performed 8 years after symptom onset shows cortical and subcortical atrophy (**a**). Sagittal FLAIR image reveals reduced size of the mesencephalic tegmentum leading to the hummingbird appearance of brainstem (**b**; red arrow). Axial FLAIR image of the brainstem shows an increased signal hyperintensity of the periaqueductal grey matter (**c**, white arrow). Furthermore, atrophy of the mesencephalic and pontine tegmentum is evident (**c**, **d**; red arrows)

Patient 1 (case 1, Table 1): A 67-year-old male complained of progressive dysphagia, diplopia, and gait instability for approximately one year, leading to admission to a neurological department. Neurological examination revealed double vision in both horizontal and upward directions along with mild limb ataxia. Brain MRI, videofluoroscopic swallow studies, and acetylcholine receptor antibodies were normal or negative. The patient was discharged with a clinical diagnosis of idiopathic dysphagia. Eight years later the patient was admitted to the Department of Neurology, Medical University of Vienna, because of hoarse voice and progressive dysphagia with aspiration of solid food. Furthermore, he complained of gait instability and recurrent falls. On admission, the patient showed a moderately broad-based and somewhat short-stepped gait, postural instability, with a positive retropulsion test, mild tremor of both hands, limb ataxia, hypometric horizontal saccades, gaze-evoked horizontal nystagmus and slowed downgaze saccades (see video, online resource). Furthermore, mild facial myokymias were observed. History revealed sleep disturbances including snoring and insomnia, nocturnal urge incontinence and signs of depression. Infectious, inflammatory, and metabolic causes were excluded by blood tests. Initial brain MRI showed mild cortical and subcortical atrophy, which progressed to mild atrophy of the corpus callosum, moderate enlargement of the third ventricle and insular cisterns and moderate midbrain atrophy during the course of the disease, suggesting a diagnosis of PSP (Fig. 1). N-omega-fluoropropyl-2beta-carbomethoxy-3beta-4-iodophenyltropan single-photon emission computed tomography (FP-CIT SPECT) revealed a clear-cut reduction of striatal dopamine transporter binding, more prominent on the left side. A ^18^F-fluorodeoxyglucose—positron emission tomography showed hypometabolism in the frontopolar and frontomesial regions. A lumbar puncture revealed clear CSF with 1 white cell per microliter, normal glucose and lactate levels, mild elevated protein (44.7 mg/dl, normal range 18-43 mg/dl) and immunoglobulins (IgG 5.16 mg/dl, normal range: 0–3.3 mg/dl; IgA 1.07 mg/d, normal range: 0–0.5 mg/dl) level. CSF oligoclonal bands were negative. A 14–3-3 assay was negative. Total tau was 273 pg/ml (normal < 450 pg/ml), phospho-tau 181P 41 pg/ml (normal < 61 pg/ml), and amyloid β 1–42 970 pg/ml (normal > 500 pg/ml). Treponema pallidum and Borrelia burgdorferi serologies in CSF were negative. Neurophysiological studies showed a normal repetitive stimulation of the facial and axillary nerves but a mixed sensorimotor polyneuropathy. Neuropsychological evaluation revealed mild cognitive impairment. During his hospital stay, the patient developed severe dysphagia and stridor resulting in hypoxemic episodes with blood oxygen levels at 60%, especially during the night requiring continuous positive airway pressure (CPAP) ventilation. A night-time apnea screening test revealed apneas of mainly central origin with an AHI (apnea-hypopnea index) of 10 (normal range: 0–5). A pneumological assessment showed hypercapnia and arterial resting hypoxemia with normal pulmonary function. Laryngoscopy revealed an impairment of vocal cord motility with decreased glottis widening during respiration, and oropharyngeal dysphagia with oral and pharyngeal retentions. Due to frequent hypoxemic episodes with massive stridor, the patient finally required tracheotomy. Overall, clinical features and ancillary investigations suggested a diagnosis of PSP. The patient was treated with levodopa, donepezil, and citalopram and discharged with a CPAP-ventilation mask. A few months later, he fell and deceased as a consequence of an extensive frontotemporal subdural haemorrhage at the age of 76 years. Five years after his death and after the first reports of IgLON5 disease, archival CSF from the patient that has been stored at -80 °C for 5 years was analysed for the presence of IgLON5 antibodies, and tested positive, leading to the retrospective diagnosis of anti-IgLON5 disease. Human leukocyte antigen testing revealed the HLA-DQB1*05:01 allele (HLA-DRB1*01:02; 03:01; HLA-DQB1*02:01; 05:01). The IgG subclass distribution and quantification were determined by flow cytometry analysis. In the CSF sample with IgLON5 antibodies, obtained 7 years after disease onset, IgG4 was the predominant subclass accounting for 80% of the total IgLON5 IgG. Serum was not available for IgLON5 antibody testing.

Patient 2 (case 7, Table 1): This 81-year-old male patient was admitted to the hospital due to acute onset of weakness of the left arm on the suspicion of a lacunar stroke. In the four preceding months, he had experienced a few imperative daytime naps per day, progressive dysphagia with significant weight loss (10 kg in 2 months, 13% of body weight, final known body weight 66 kg), and dysarthria. Moreover, he showed chorea of the upper limbs, with left predominance. Previous history included coronary heart disease, metabolic syndrome including diabetes and hypercholesterolemia and resection of a vestibular schwannoma. Generalized atrophy, but no acute vascular lesions were evident on brain MRI. EEG showed generalized slowing, but no interictal or ictal discharges. A polysomnography showed a significantly elevated AHI (apnea-hypopnea index) of 61. During the hospital stay, central sleep apnea with severe hypoxemic episodes became evident. A whole-body PET CT ruled out a neoplastic process. CSF levels of 14–3-3 protein, were within the normal range and real-time Quaking-induced conversion assay (RTQuIC) for prion protein detection was negative. Subsequent testing for autoantibodies revealed positive IgLON5 antibodies in serum and CSF. The antibody subclass distribution was IgG4 96% to IgG1 4% in CSF and IgG4 90% to IgG1 10% in serum. The patient received 0.4 g/kg IV immunoglobulins (IVIG) for 5 days (infusion speed 0.3 ml/kg over the first 30 min, followed by stepwise increase with a maximum infusion speed of 4.8 ml/kg per hour). A remarkable improvement of dysarthria was noted after two days of IVIG but this was followed by rapid deterioration resulting in carbon dioxide narcosis after finishing the IVIG cycle. In addition, the patient also suffered from pneumonia, which worsened the respiratory situation. Treatment escalation with rituximab (total dosage 663.8 mg (375 mg/m^2^)) was started. However, 5 days later the patient died from pneumonia and sepsis.

Patient 3 (case 9, Table 1): This female patient presented to the sleep-laboratory first time at the age of 67 years because of disturbing snoring, but at that time did not follow the recommendation to undergo polysomnography. Five years later, at the age of 72 years she presented again with insomnia and dysphagia. A polysomnography showed stridor, REM sleep behavior disorder, and minor hand and finger movements as well as talking during the transition to sleep and N1. Because of this constellation, anti-IgLON5 disease was suspected and anti-IgLON5 antibodies were confirmed in serum and CSF. Additionally, moderate obstructive sleep apnea was present with AHI 26.6/h and 18/h (first and second night), but only mild desaturations (10.2 and 8.4/h). CPAP was initiated, also with the aim to improve the stridor. Dysarthria was noted and increased in the following years. Balance problems followed and progressed during the following two years resulting in recurrent falls. As dysphagia worsened a percutaneous gastrostomy was implanted. During the disease course, treatment included two cycles of IVIG (30 g once daily for 5 days), two cycles of high-dose methylprednisolone, one cycle of rituximab (1 g each) and one cycle of plasmapheresis (4 days). Unfortunately, the patient subjectively and objectively did not benefit from treatment. She developed a marked weight loss in the last years (13 kg in 3 years, 20% of body weight, last known body weight 52 kg), despite percutaneous gastrostomy. Additional autonomic failure, in the form of transient orthostatic hypotension and severe gait instability further worsened the patient’s condition. The patient declined tracheostomy and was referred to palliative care, and died from global respiratory failure at the age of 76.

### Neuropathology

Neuropathological investigation of the nine brain autopsies (eight definite, one probable) showed a variable combination of tau pathology and inflammation.

**Fig. 2:**
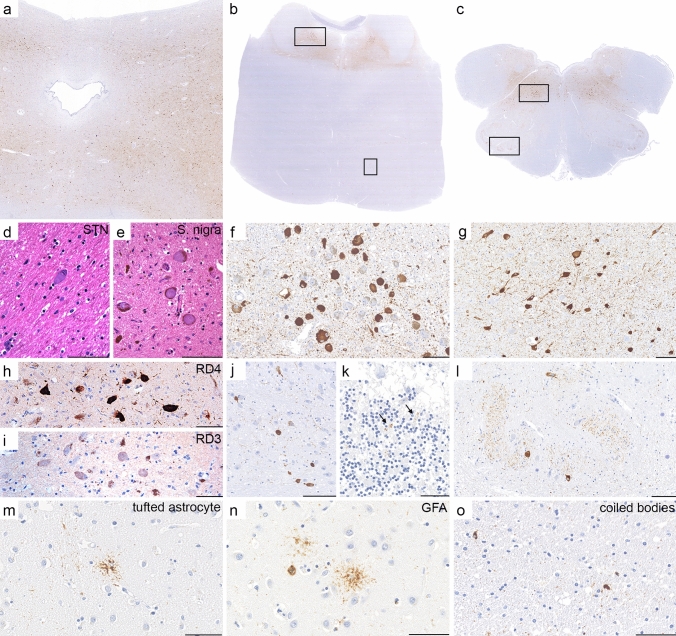
PSP neuropathology in anti-IgLON5 disease (patient 1). Neuropathology shows neuronal loss and the presence of neurofibrillary tangles in the tegmentum of the midbrain, pons, and medulla oblongata (**a**–**c**; AT8), subthalamic nucleus (**d**; H&E), substantia nigra pars compacta (**e**; H&E), locus coeruleus (**f**; AT8; upper rectangle in **b** enlarged in **f**), and magnocellular nuclei of the medulla oblongata (**g**; AT8, upper rectangle in **c** enlarged in **g**) that were strongly immunoreactive for hTau, particularly for 4-repeat tau isoforms (**h**, substantia nigra; RD4) and negative for 3-repeat tau isoforms (**i**, substantia nigra; RD3). In addition, neurofibrillary tangles and neuropil threads were visible in the pontine base (**j**; AT8; lower rectangle in b enlarged in **j**), synaptic glomeruli of the cerebellar cortex (**k**; AT8; arrows), and olivary nucleus (**l**; AT8; lower rectangle in c enlarged in **l**). Glial pathology in basal ganglia consists of tufted astrocytes (**m**; AT8), some granular fuzzy astrocytes (**n**; GFAP), and oligodendroglial coiled bodies (**o**; AT8). Scale bars: **d**, **e**, **k**, **m**, **n**, o: 50 μm; **f**–**j**, l: 100 μm

Tau pathology (Table 2): Five cases (55.5%, four previously published) showed the already described 3R-tau and 4R-tau pathology with tau-positive neuronal inclusions mainly in hypothalamus and tegmentum of the brainstem, according to the neuropathological criteria of anti-IgLON5-related tauopathy [11]. Three cases (2, 3, and 7) showed features of age-related neurofibrillary pathology in the limbic system and variable β-A4-amyloid deposits (mild to moderate plaques and mild amyloid beta angiopathy), but not the classical IgLON5-related brainstem tauopathy. Finally, one of the newly identified patients (case 1) was re-evaluated from the neuropathology archives 5 years after death. Archival paraffin blocks were available from basal ganglia, thalamus, midbrain (Fig. 2a), pons (Fig. 2b), and medulla oblongata (Fig. 2c). We found a prominent neuronal and glial tauopathy, characterized by abundant large neurofibrillary tangles of the globose type in large cholinergic neurons of the basal ganglia, in the subthalamic nucleus (Fig. 2d), in the substantia nigra (Fig. 2e), in the locus coeruleus (Fig. 2f), and in the magnocellular nuclei of the medulla oblongata (Fig. 2g). These areas showed prominent neuronal loss. Neurofibrillary tangles were strongly immunoreactive for hyperphoshorylated tau (AT8) and were mainly composed of 4R-tau isoforms (Fig. 2h, i). Neurofibrillary tangles and threads were also detected in the midbrain tectum (Fig. 2a), in the pontine base (Fig. 2j), cerebellar cortex (Fig. 2k) and in the inferior olives (Fig. 2l). The glial tau pathology was characterized by tufted astrocytes that were preferentially observed in the caudate nucleus (Fig. 2m) and some granular fuzzy astrocytes (GFA; Fig. 2n). Oligodendroglial coiled bodies (Fig. 2o) were also observed in the basal ganglia and brainstem regions. These features were consistent with those described in brainstem-predominant PSP (Richardson Syndrome). Immunohistochemistry for 3Rtau was negative (Fig. 2i). Amyloid plaques were absent.

**Table 2:** Description of neuropathology/tau-pathology in nine patients with anti-IgLON5 disease

Patient	Tau-pathology
1	Brainstem predominant PSP Neuronal and glial tauopathy in the thalamus, tegmentum and the basis of the brainstem, 4R tau positive tangles and neuropil threads, coiled bodies and tufted astrocytes, compatible with PSP, 3R tau was negative. Amyloid plaques were absent. pTDP43 inclusions absent
2 [4]	Age-related neurofibrillary pathology Neuropil threads and sparse neuronal tangles in dorsomedial and ventromedial nuclei. Also, in Locus coeruleus, mixture of 3R and 4R isoforms, neurofibrillary tangles, pre-tangles and neuropil threads in all hippocampal regions, especially CA2 and entorhinal cortex. Amyloid beta absent, α-synuclein absent, pTDP43: neuronal skein-like and granular cytoplasmic inclusions in the thalamus, striatum, midbrain, also in parenchymal ramified microglial cells and in perivascular microglia in the thalamus, hippocampus, striatum, nucleus basalis, midbrain
3 [5]	Age-related neurodegenerative pathology Neurofibrillary pathology and β-amyloid deposits consistent with Alzheimer’s disease of intermediate severity (Braak neurofibrillary stage III, Thal phase 4, CERAD plaque score B; Alzheimer´s disease neuropathological changes A3,B2,C2)
4 [14]	Classical IgLON5-related brainstem tauopathy Prominent tauopathy (4R and 3R) characterized by numerous Gallyas-positive neurofibrillary tangles, diffuse granular cytoplasmic phospho-tau (pTau; AT8) immunoreactivity (pre- tangles), and neuropil threads involving predominantly the hypothalamus, zona incerta, hippocampus, tegmentum of the brainstem (mesencephalon, pons, and medulla), and cervical spinal cord
5 [24]	Classical IgLON5-related brainstem tauopathy pTau (AT8) pathology in the hippocampus, entorhinal cortex, N. basalis Meynert, substantia innominata, septal nuclei, hypothalamus, in the brainstem, isolated pTau pathology in the ant. cingulate cortex, amygdala, pallidum, thalamus, subthalamic nucleus, substantia nigra, dentate nucleus, cervical spinal cord. 3R and 4R tau isoforms present
6 [24]	Classical IgLON5-related brainstem tauopathy pTau (AT8) pathology in the hippocampus, entorhinal cortex, amygdala, N. basalis Meynert, substantia innominata, zona incerta, subthalamic nucleus, hypothalamus, brainstem, isolated pTau pathology in the striatum, pallidum, ant. cingulate cortex, thalamus, corpus mamillare, substantia nigra, dentate nucleus, cervical spinal cord. 3R and 4R tau isoforms present. pTDP43: some neuronal skein-like, small compact or diffuse granular inclusions in neurons of the brainstem and spinal cord
7	Age-related neurodegenerative pathology Neurofibrillary pathology restricted to limbic system (Braak III), few neuritic plaques (CERAD Score B) and Thal phase 4 (Alzheimer´s disease neuropathological changes A3,B2,C2), moderate vascular amyloidosis, ARTAG, granular fuzzy astrocytes, few Lewy-type/alpha-synuclein neurites in the olfactory system (olfactory-only)
8 [22]	Probable anti-IgLON5 disease—classical IgLON5-related brainstem tauopathy pTau (AT8) pathology in the hippocampus, entorhinal cortex, amygdala, N. basalis Meynert, substantia innominata, zona incerta, hypothalamus, brainstem, cervical spinal cord; isolated tau pathology in the subthalamic nucleus, thalamus, striatum, corpus mamillare. 3R and 4R tau isoforms present
9	Classical IgLON5-related brainstem tauopathy pTau (AT8) pathology in the hypothalamus, brainstem tegmentum, medulla oblongata with neuronal cell loss, gliosis, microglia activation with tau positive tangles and neurites. 3R and 4R tau isoforms present. Mild brainstem predominant Lewy body/alpha-synuclein pathology; small vessel disease

Other proteinopathies: Concomitant with the tau pathology we found transactive response DNA-binding protein of 43 KDa (pTDP-43) inclusions in neurons of the brainstem, anterior horn neurons of the spinal cord and microglia of cases 2 and 6 (previously reported). Topographical and cellular distribution of pTDP-43 pathology differed from that of limbic-predominant age-related TDP-43 encephalopathy (LATE) or frontotemporal lobar degeneration (FTLD), with some reminiscence to ALS morphology and distribution, but would currently fall within the spectrum of unusual TDP-43 pathology [20]. Focal incidental Lewy-body type alpha-synuclein aggregates were identified in some brainstem nuclei (case 9) and in the olfactory bulb (case 7) (brainstem predominant and olfactory only Lewy-type pathology according to the new consensus criteria, respectively [2].

**Fig. 3:**
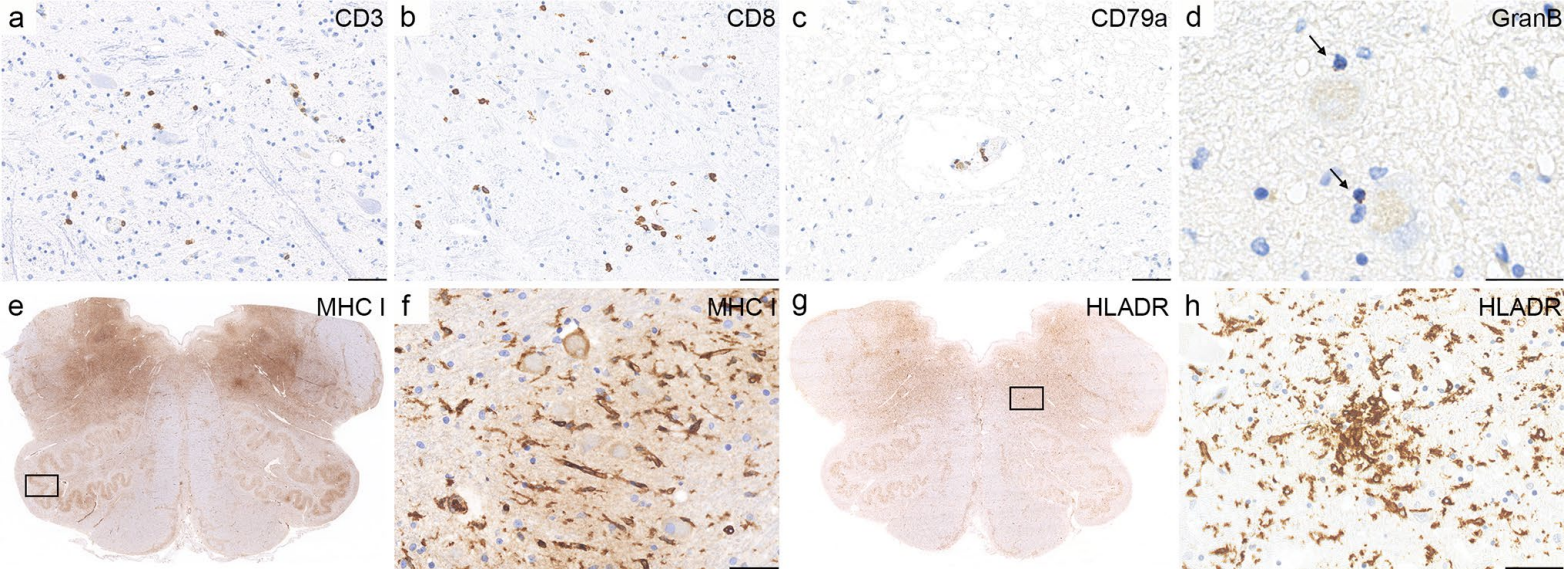
Cellular inflammation in anti-IgLON5 disease. Cellular inflammation was mild to moderate and mainly composed of perivascular and parenchymal CD3 (**a**) and CD8 positive T cells (**b**) and few perivascular CD79a positive B cells/plasma cells (**c**). Parenchymal CD8 T cells were granzyme B positive and granules showed a polarization towards neurons (**d**; arrows). In addition, neurons showed an upregulation of MHC class I in the reticular formation and olivary nuclei (**e**, rectangle enlarged in **f**). Marked microglia activation was found in the HLA-DR staining in the tegmentum of medulla oblongata and nucleus olivaris (**g**), including the formation of microglial nodules (**h**; rectangle in g enlarged in h). Images are depicted from patient 1. Scale bars: 50 μm

**Fig. 4:**
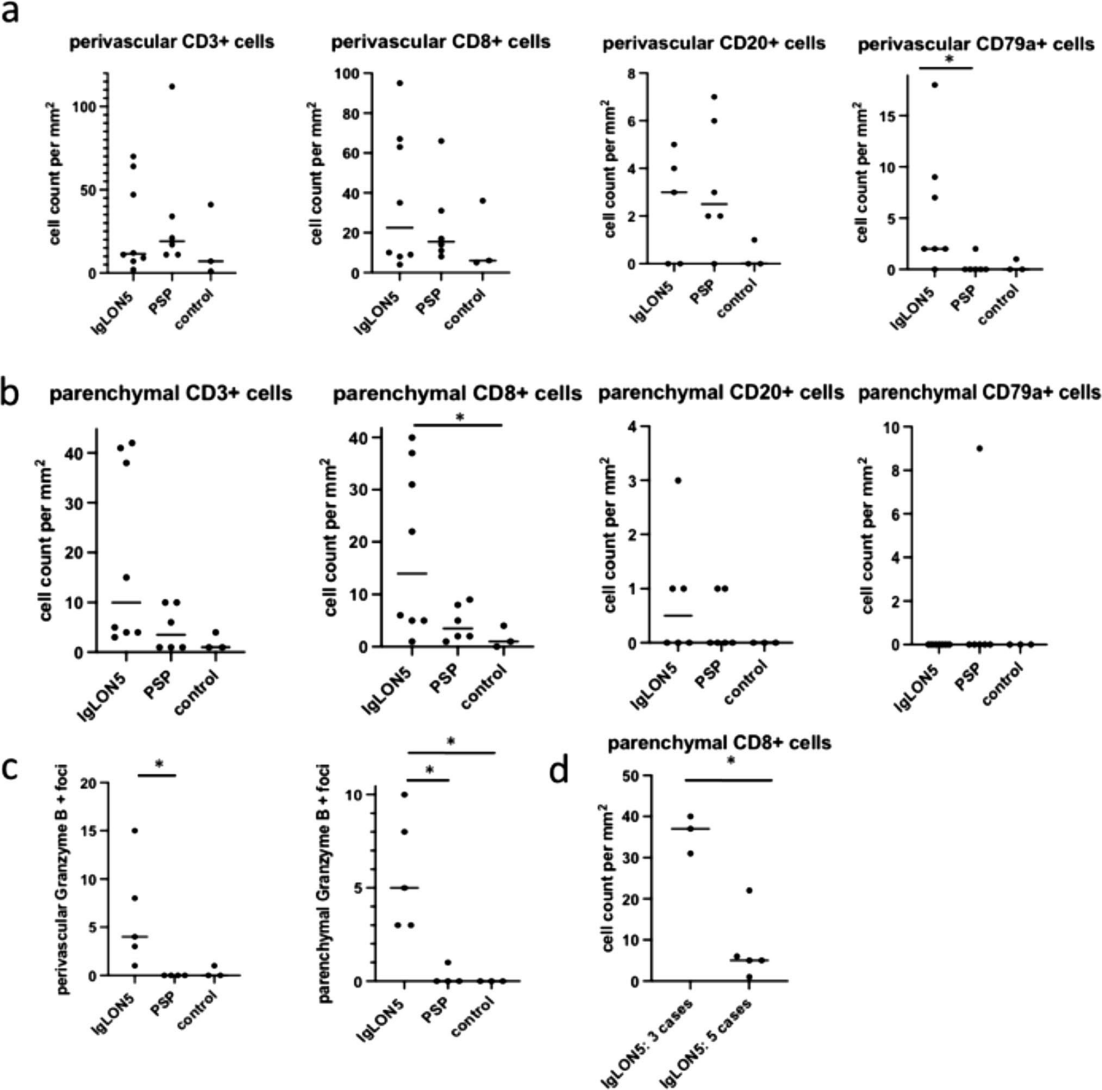
Inflammation in anti-IgLON5 disease. Scatter dot plots with an overview of CD3 + , CD8 + T cells and CD20 + , CD79a + B-/plasma cells either perivascular (**a**) or in the parenchyma (**b**) of the brainstem of definite anti-IgLON5 patients. CD8/Granzyme B + T cells were significantly more frequent in the parenchyma of IgLON5 patients (IgLON5 vs. PSP *p* = 0.03; IgLON5 vs. control *p* = 0,01) (**c**). Three cases showed significantly (*p* = 0.04) more parenchymal CD8 + T cells among them two cases with short disease duration and no tauopathy and one case with PSP-like pathology but no immunotherapy compared to the other IgLON5 patients (**d**). A non-parametric Kruskal–Wallis or Mann–Whitney test has been performed, using a post-hoc Dunn`s multiple comparison correction, *can be assumed as *p* < 0.05 if not otherwise stated, not significant differences are not illustrated

Cellular inflammation: Inflammatory infiltrates in the eight definite anti-IgLON5 cases were mild to moderate and mainly composed of perivascular and parenchymal CD3 and CD8 positive T cells and few perivascular CD20 and CD79a positive B cells/plasma cells (Supplementary Table 1, online resource; Fig. 3a–c). No expansion of CD20 or CD79a positive B cells was observed in meninges, perivascular compartments, or parenchyma (other B cell markers were not investigated). Quantification of perivascular CD3/CD8 + T cells in the tegmentum of the brainstem (region of tau-pathology) did not differ between anti-IgLON5 disease and PSP without anti-IgLON5 antibodies, however parenchymal CD8/granzyme B + T cells were significantly more frequent in anti-IgLON5 patients (granzyme B staining available in 5 cases) compared to PSP and controls (Fig. 4a–c). The granzyme B + granules showed a polarization towards neurons (Fig. 3d), in addition, we found an upregulation of MHC class I molecules on neurons in the reticular formation and olivary nucleus (Fig. 3e, f). In further analysis of five cases with sections available, some perivascular and parenchymal lymphocytes showed an expression of PD1 (data not shown). Marked microglia activation was found in the HLA-DR staining in the tegmentum and basis of the brainstem, nucleus olivaris, cerebellar cortex, and CA1-CA4 in hippocampus areas including the formation of microglial nodules (Fig. 3g, h). Inflammatory infiltrates could not be evaluated in patient 8, [22] who presented a tauopathy suggestive of anti-IgLON5 disease but IgLON5 antibodies could not be evaluated.

**Fig. 5:**
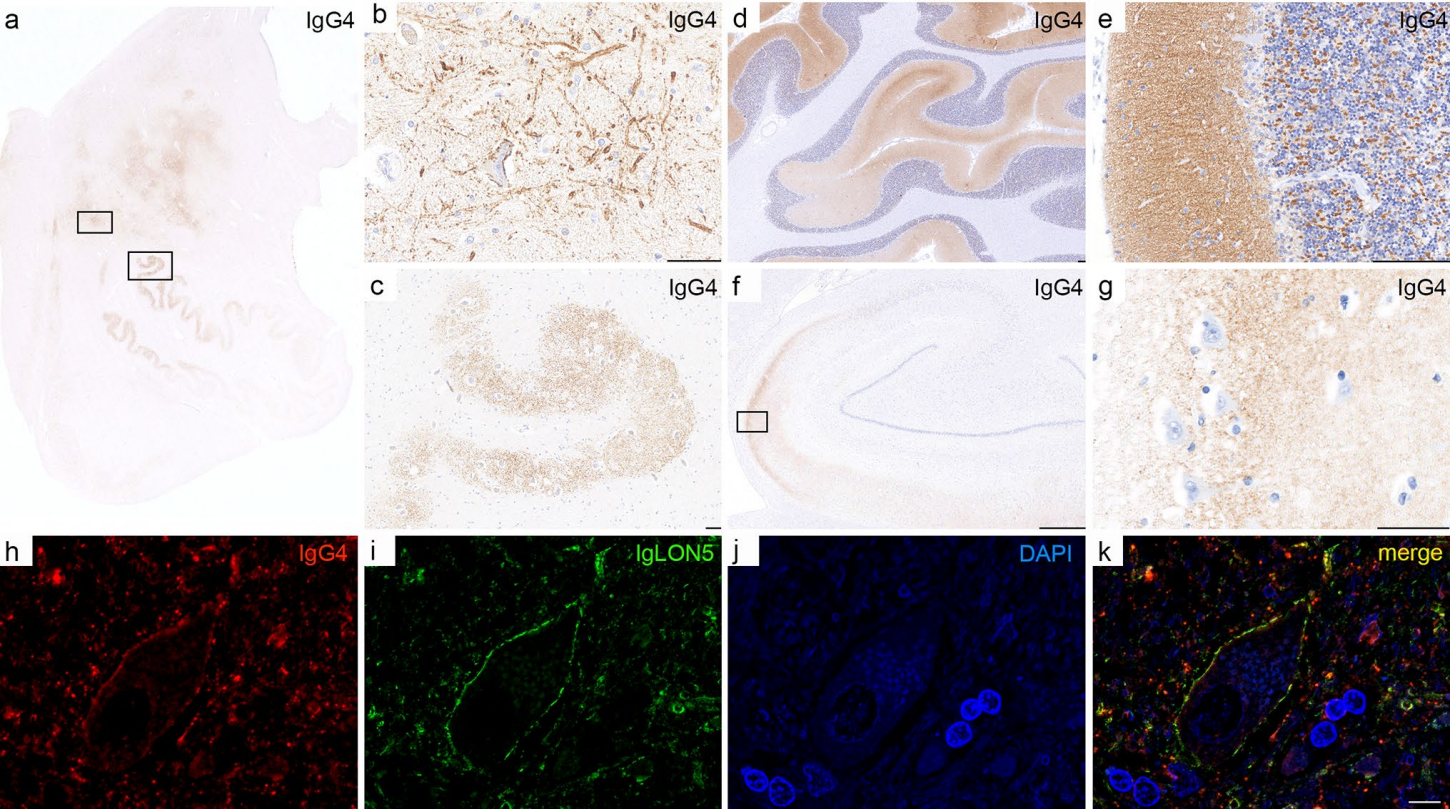
IgG4 deposits in anti-IgLON5 disease. IgG4 deposits were prominent in the tegmentum of the brainstem and olivary nucleus (**a**–**c**; rectangles in an enlarged in **b** and **c**), molecular layer and synaptic glomeruli in the granule cell layer of the cerebellar cortex (**d**, **e**), and one case also showed prominent IgG4 deposition in the CA1-CA2 sector of the hippocampus (**f**, **g**; rectangle in **f **enlarged in **g**). Immunofluorescence double labelling revealed a co-localisation of IgG4 deposits (**h**, red) with IgLON5 (**i**, green) on neuronal membranes and the neuropil (**j**, DAPI; **k**, merge). Images **a**-**c** and **h**–**k** are depicted from patient 3, and images **d**-**g** are depicted from patient 7; Scale bars: **b**, **c**, **g**: 50 μm; **d**, **e**: 150 μm; **f**: 1 mm; **k**: 10 μm

Humoral inflammation: Deposition of IgG, IgG1, IgG4, and terminal complement complex C9neo was investigated in eight cases (seven definite, one probable, Supplementary Table 2, online resource). We found a strong and specific deposition of IgG4 in the neuropil and on the membrane of neuronal cell bodies that was most prominent in cases 3 and 7 and involved the tegmentum of the brainstem, olivary nucleus, cerebellar cortex (molecular layer and synaptic glomeruli in the granule cell layer), and pontine base (Fig. 5a–e). One case (7) also showed prominent IgG4 deposition in the CA1-CA2 sector of the hippocampus (Fig. 5f, g) and one of three available spinal cord sections showed mild IgG4 deposition in the posterior horn (data not shown). Double immunofluorescence revealed a co-localisation of IgG4 deposits with IgLON5 on neuronal membranes and within the neuropil (Fig. 5h–k). Areas with prominent IgG4 deposition showed reactive gliosis (data not shown) but neurons with IgLON5 immunoreactivity were still preserved (Fig. 5i). Mild neuropil IgG1 deposits were found in the cerebellar cortex in areas with IgG4 deposition in four cases, but other areas were negative (Supplementary Table 2, online resource). Activated complement deposition (C9neo) was not found. No IgG1 or IgG4 deposition was detectable in six seronegative PSP autopsies, one sepsis, and one acute hypoxic brain damage. An anti-NMDAR encephalitis case showed strong deposition of IgG1 mainly in the hippocampus and hypothalamus, IgG4 was negative. Humoral inflammation could not be evaluated in case 2 due to a lack of tissue [4].

### Correlation of tau pathology and inflammation with HLA phenotype and disease duration

The HLA phenotype was available in eight anti-IgLON5 cases. The classical risk HLA haplotype DRB1*10:01 and DQB1*05:01 did not associate with the IgLON5 tauopathy as it was present in three patients with and two without the classical anti-IgLON5-related tauopathy. Disease duration was available in the nine anti-IgLON5 disease cases. The three patients with age-related tau-pathology had a documented median disease duration of 1.25 years (range 0.5–2 years) while those patients with classical anti-IgLON5-related tauopathy or PSP with prominent brainstem involvement had a median disease duration of 9 years (range 0.5–13 years) (*p* = 0.11).

Cellular inflammation (T cells and B cells) was mild to moderate and did not correlate with the HLA phenotype, tau pathology, or disease duration. However, there were three cases that showed significantly more parenchymal CD8 + T cells compared to the other six IgLON5 cases (Fig. 4d). One of them was the patient with PSP pathology (case 1), who had a long disease duration (13 years) but did not receive immunotherapy, the other two cases (2, 3) received immunotherapy but had a short disease duration and no brainstem tauopathy (see Tables 1 and 2). The two cases with the most prominent IgG4 deposits (3 and 7) had a shorter disease duration (6 and 24 months) compared to the six cases with less prominent or even absent IgG4 deposition with a median disease duration of 9 years; range: 0.5–13 years (p > 0.05, not significant). Both cases with abundant IgG4 deposition carried two HLA risk alleles and showed only a temporary or no response to IVIG and died shortly after the start of therapy.

## Discussion

In this retrospective neuropathological study, we investigated a postmortem cohort of nine patients with anti-IgLON5 disease. The previously described brainstem neuronal tauopathy was observed in five patients, who had a long disease duration (median 9 years), whereas pronounced IgG4 deposition was seen in two patients with shorter disease duration (median 1.25 years) and without the typical IgLON5-related brainstem tauopathy. In addition, we identified one patient, who was diagnosed retrospectively as anti-IgLON5 disease and had a brainstem tauopathy; however, neuropathological alterations fulfilled criteria of PSP, a constellation not described so far for anti-IgLON5 disease. This finding expands the spectrum of anti-IgLON5-related tauopathy.

The few existing neuropathological studies on anti-IgLON5 disease have focused on the identification and characterisation of the type and distribution of tau-pathology and only briefly mentioned inflammatory findings [4, 5, 11, 24]. The aim of our study was to investigate in more detail the role of cellular and humoral factors involved in the pathophysiology of anti-IgLON5 disease in an unselected cohort of autopsies with a range of clinical and neuropathological phenotypes, including cases with and without the expected brainstem tauopathy [7, 9, 24]. One of our main immunological findings is that two patients with anti-IgLON5 disease without brainstem tauopathy had an intense deposition of IgG4 in the neuropil and along the membrane of neurons in the tegmentum of the brainstem, olivary nuclei, and cerebellar cortex that co-localized with IgLON5. One case also showed IgG4 deposits in the CA1-CA2 sector of the hippocampus. These areas have been previously described as predilection sites for anti-IgLON5-related tauopathy. [11, 24]. The prominent IgG4 deposition in these patients might reflect a specific disease pattern of anti-IgLON5 disease and could indicate that immune mechanisms antedate the tau pathology in anti-IgLON5 disease. Interestingly, patients with prominent IgLON5-related brainstem tauopathy (with variable disease duration) showed less IgG4 deposits. This could result from loss of synaptic neuronal function with less IgLON5 activity (although IgLON5 immunoreactivity was still preserved) and/or be related to immunotherapy, but probably more cases have to be tested to confirm this association.

Anti-IgLON5 disease patients harbour both IgG1 and IgG4 antibodies in the serum and CSF, with IgG4 being more abundant [9, 21, 25]. The potential pathogenic mechanisms of these two anti-IgLON5 autoantibody subclasses are different. Previous neuronal culture experiments revealed that IgG1 autoantibodies can trigger the internalization of IgLON5 while IgG4 does not. In a subsequent experiment, total IgLON5 IgG resulted in abnormal accumulation of neurofilaments in neurons, however, the role of specific IgG subclasses was not evaluated [17, 25]. In contrast, both IgLON5 IgG1 and IgG4 antibodies can block the protein interaction of soluble IgLON5 with IgLON family members 1 to 5, present in the membrane of transfected HEK cells and in rat hippocampal neurons [18]. The IgG4 deposits in our study co-localized with IgLON5 along the neuronal membranes, supporting the interaction between IgG4 antibodies and IgLON5 in humans. However, how the blocking of IgLON5 interferes with downstream signal transduction pathways that may result in tauopathy is still unclear. Interestingly, deposition of IgG1 was less well detectable by immunohistochemistry, possibly reflecting the lower ratio of IgG1 in patients and/or the different interaction with IgLON5 that results in the internalization of the protein [25].

The neuroanatomical distribution of IgG4 deposits in the two patients without IgLON5-related brainstem tauopathy correlated well with their clinical phenotype with prominent brainstem manifestations, which supports that an immune-mediated mechanism contributes to neuronal dysfunction. Interestingly, neurons appeared still preserved in areas of intense IgG4 deposition and did not show a reduced IgLON5-immunoreactivity. This might indicate a reversible effect of anti-IgLON5 IgG4 and could explain why a subgroup of patients shows at least a partial clinical response to immunotherapy [13, 21]. A possible pathophysiological cascade could start with the deposition of anti-IgLON5 IgG4 antibodies at the surface of neurons that results in synaptic neuronal dysfunction (immunotherapy effective state). In a second step, IgG1 antibodies cause an internalization of IgLON5 that results in tau aggregation, neuronal death, and neurodegeneration. Our findings therefore also support the hypothesis that early start of immunotherapy such as IVIG, plasma exchange and/or B cell depletion by rituximab might be beneficial for at least a subgroup of patients and could prevent the progression of disease. However, current data are too preliminary and future clinical studies will be necessary to provide treatment recommendations.

In addition to humoral immune mechanisms, we found pronounced microglia activation including microglial nodules and some mild T cell-mediated pathology. This was characterized by the upregulation of MHC class I molecules on neurons and CD8 + /granzyme B + cytotoxic T cells in the parenchyma of the brainstem. Although the T cell-mediated inflammation did not appear to be the dominant pathology, it might have contributed to ongoing neurodegeneration and cell death. However, a limitation of our study is that most patients received immunotherapy before death which might have influenced the intensity of inflammation. In addition, the cause of death, particularly infections in the latest disease stages, may alter the brain microenvironment. Moreover, it is known that in general neurodegenerative disorders, including tauopathies and synucleinopathies, may also show some T cell infiltrates in affected areas, at least at early disease stages [1, 10, 27]. None of our cases showed C9neo deposits. The lack of activated terminal complement complex can also be observed in other forms of anti-neuronal autoimmune encephalitis such as anti-NMDAR encephalitis [28]. However, this does not exclude a role of complement components in the pathogenesis of the disease and future studies will be necessary to investigate, which innate immune factors are involved in driving chronic inflammation and neurodegeneration in anti-IgLON5 disease.

One of our cases showed neuropathological characteristics of PSP. This case was retrospectively identified to have IgLON5 antibodies and the clinical work-up was performed at a time when anti-IgLON5 disease was not known. At that time the patient was peculiar because of an atypical clinical presentation. Disease course and clinical features including early falls, hypometric horizontal saccades and slowed downgaze saccades were suggestive of PSP and dopaminergic degeneration was confirmed by dopamine transporter imaging. However, the case also showed substantial peculiarities including prominent bulbar symptoms, severe dysphagia with vocal cord paresis, and nocturnal stridor with severe hypoxemic episodes requiring CPAP. The massive stridor and the resulting hypoxemic episodes were the dominant and most limiting factors during his hospital stay and cannot be explained by PSP. Retrospectively, these symptoms are also compatible with anti-IgLON5 disease [9]. The originally described anti-IgLON5-related tauopathy has been defined as mostly restricted to neurons and characterized by a mixture of 3R and 4R tau pathology [11]. Interestingly, the post-mortem neuropathological features of our patient fulfilled the criteria of PSP, a 4-repeat tauopathy that involved cortical and subcortical structures and affects neurons, astrocytes, and oligodendrocytes. The distribution of PSP pathology, particularly in the brainstem, has overlapping features with the classical anti-IgLON5-related tauopathy and has to be considered in the differential diagnosis [24]. However, an important difference is the glial involvement, particularly tufted astrocytes that define the pathology of PSP independently of the topographical extension and severity of neuronal tau pathology [23]. In our patient, only the affection of the synaptic glomerula of the cerebellar cortex was unusual for PSP and suggests an overlap with classical anti-IgLON5 related tauopathy, where this feature has been reported.

The reason for the 4R tau-only pathology involving also glial cells in our patient is unclear and factors such as genetic risk variants e.g., microtubule-associated protein tau (MAPT) genotype, disease duration, age of the patient, or several immunological mechanisms e.g. distinct HLA alleles might have altered the neuropathological pattern [8]. Neuropathological studies of more cases are definitely necessary to be able to identify and associate specific risk factors that may influence or modulate the development of a specific type of tauopathy.

Previous studies investigated whether IgLON5 antibodies should be routinely tested in patients with clinical presentation of PSP. A study screening for IgLON5 antibodies among a cohort of 32 patients with a clinical diagnosis of PSP found one positive patient, other studies turned out to be negative [12, 19, 24]. We would recommend testing for IgLON5 antibodies only in cases of suspected PSP, which have atypical clinical features, such as stridor, dysphagia, or parasomnias, that are uncommon in PSP patients [15, 19, 24].

## Conclusion

Our data suggest that neuropathology of anti-IgLON5 disease is time dependent and tau pathology tends to occur in later disease stages. The tau pathology predominantly affects the brainstem and cerebellum and is mostly characterized by a combination of 4R and 3R tau pathology restricted to neurons but may also present a PSP-phenotype with neuronal and glial 4R tau pathology. Two patients with a short disease duration and severe clinical phenotype of anti-IgLON5 disease showed early and prominent deposition of anti-IgLON5 IgG4 at predilection sites for tau pathology, but without tau accumulation. Whether this reflects a specific neuropathological pattern in anti-IgLON5 disease and indicates that anti-IgLON5 antibodies precede the tau pathology needs to be investigated in future experimental studies. Early start of immunotherapy may prevent irreversible neuronal damage and progression of the disease.

## Supplementary Information

Below is the link to the electronic supplementary material.

Supplementary file 1 (DOCX 41 KB)

Supplementary file 2 (MOV 18591 KB)

## Data Availability

Data can be made available from the corresponding authors on reasonable request and after approval from the ethics review board at the Medical University of Vienna.

## References

[1] Altendorfer B, Unger MS, Poupardin R, Hoog A, Asslaber D, Gratz IK (2022). Transcriptomic profiling identifies CD8(+) t cells in the brain of aged and Alzheimer’s Disease transgenic mice as tissue-resident memory T cells. J Immunol.

[2] Attems J, Toledo JB, Walker L, Gelpi E, Gentleman S, Halliday G (2021). Neuropathological consensus criteria for the evaluation of Lewy pathology in post-mortem brains: a multi-centre study. Acta Neuropathol.

[3] Bruggemann N, Wandinger KP, Gaig C, Sprenger A, Junghanns K, Helmchen C (2016). Dystonia, lower limb stiffness, and upward gaze palsy in a patient with IgLON5 antibodies. Mov Disord.

[4] Cagnin A, Mariotto S, Fiorini M, Gaule M, Bonetto N, Tagliapietra M (2017). Microglial and neuronal TDP-43 pathology in Anti-IgLON5-related tauopathy. J Alzheimers Dis.

[5] Erro ME, Sabater L, Martinez L, Herrera M, Ostolaza A, Garcia de Gurtubay I (2020). Anti-IGLON5 disease: A new case without neuropathologic evidence of brainstem tauopathy. Neurol Neuroimmunol Neuroinflamm.

[6] Gaig C, Compta Y (2019). Neurological profiles beyond the sleep disorder in patients with anti-IgLON5 disease. Curr Opin Neurol.

[7] Gaig C, Compta Y, Heidbreder A, Marti MJ, Titulaer MJ, Crijnen Y (2021). Frequency and characterization of movement disorders in Anti-IgLON5 disease. Neurology.

[8] Gaig C, Ercilla G, Daura X, Ezquerra M, Fernandez-Santiago R, Palou E (2019). HLA and microtubule-associated protein tau H1 haplotype associations in anti-IgLON5 disease. Neurol Neuroimmunol Neuroinflamm.

[9] Gaig C, Graus F, Compta Y, Hogl B, Bataller L, Bruggemann N (2017). Clinical manifestations of the anti-IgLON5 disease. Neurology.

[10] Galiano-Landeira J, Torra A, Vila M, Bove J (2020). CD8 T cell nigral infiltration precedes synucleinopathy in early stages of Parkinson’s disease. Brain.

[11] Gelpi E, Hoftberger R, Graus F, Ling H, Holton JL, Dawson T (2016). Neuropathological criteria of anti-IgLON5-related tauopathy. Acta Neuropathol.

[12] Giannoccaro MP, Gastaldi M, Rizzo G, Jacobson L, Vacchiano V, Perini G (2021). Antibodies to neuronal surface antigens in patients with a clinical diagnosis of neurodegenerative disorder. Brain Behav Immun.

[13] Gruter T, Mollers FE, Tietz A, Dargvainiene J, Melzer N, Heidbreder A (2023). Clinical, serological and genetic predictors of response to immunotherapy in anti-IgLON5 disease. Brain.

[14] Hogl B, Heidbreder A, Santamaria J, Graus F, Poewe W (2015). IgLON5 autoimmunity and abnormal behaviours during sleep. Lancet.

[15] Honorat JA, Komorowski L, Josephs KA, Fechner K, St Louis EK, Hinson SR (2017). IgLON5 antibody: Neurological accompaniments and outcomes in 20 patients. Neurol Neuroimmunol Neuroinflamm.

[16] Koneczny I, Cossins J, Waters P, Beeson D, Vincent A (2013). MuSK myasthenia gravis IgG4 disrupts the interaction of LRP4 with MuSK but both IgG4 and IgG1–3 can disperse preformed agrin-independent AChR clusters. PLoS ONE.

[17] Landa J, Gaig C, Plaguma J, Saiz A, Antonell A, Sanchez-Valle R (2020). Effects of IgLON5 Antibodies on Neuronal Cytoskeleton: A Link between Autoimmunity and Neurodegeneration. Ann Neurol.

[18] Landa J, Serafim AB, Gaig C, Saiz A, Koneczny I, Hoftberger R (2023). Patients’ IgLON5 autoantibodies interfere with IgLON5-protein interactions. Front Immunol.

[19] Mangesius S, Sprenger F, Hoftberger R, Seppi K, Reindl M, Poewe W (2017). IgLON5 autoimmunity tested negative in patients with progressive supranuclear palsy and corticobasal syndrome. Parkinsonism Relat Disord.

[20] Nelson PT, Lee EB, Cykowski MD, Alafuzoff I, Arfanakis K, Attems J (2023). LATE-NC staging in routine neuropathologic diagnosis: an update. Acta Neuropathol.

[21] Nissen MS, Blaabjerg M (2019). Anti-IgLON5 disease: a case with 11-year clinical course and review of the literature. Front Neurol.

[22] Pretnar-Oblak J, Zaletel M, Hajnsek TM, Meglic B, Hocevar-Boltezar I, Popovic M (2010). Isolated bulbar paralysis in a patient with medullar tau pathology: a case report. J Neurol Neurosurg Psychiatry.

[23] Roemer SF, Grinberg LT, Crary JF, Seeley WW, McKee AC, Kovacs GG (2022). Rainwater Charitable Foundation criteria for the neuropathologic diagnosis of progressive supranuclear palsy. Acta Neuropathol.

[24] Sabater L, Gaig C, Gelpi E, Bataller L, Lewerenz J, Torres-Vega E (2014). A novel non-rapid-eye movement and rapid-eye-movement parasomnia with sleep breathing disorder associated with antibodies to IgLON5: a case series, characterisation of the antigen, and post-mortem study. Lancet Neurol.

[25] Sabater L, Planaguma J, Dalmau J, Graus F (2016). Cellular investigations with human antibodies associated with the anti-IgLON5 syndrome. J Neuroinflammation.

[26] Stam NJ, Vroom TM, Peters PJ, Pastoors EB, Ploegh HL (1990). HLA-A- and HLA-B-specific monoclonal antibodies reactive with free heavy chains in western blots, in formalin-fixed, paraffin-embedded tissue sections and in cryo-immuno-electron microscopy. Int Immunol.

[27] van Olst L, Coenen L, Nieuwland JM, Rodriguez-Mogeda C, de Wit NM, Kamermans A (2022). Crossing borders in Alzheimer‘s disease: A T cell’s perspective. Adv Drug Deliv Rev.

[28] Zrzavy T, Endmayr V, Bauer J, Macher S, Mossaheb N, Schwaiger C, Ricken G, Winklehner M, Glatter S, Breu M (2021). Neuropathological Variability within a Spectrum of NMDAR-Encephalitis. Ann Neurol.

