# Supplementary material for: Analysis of inflammatory markers and tau deposits in an autopsy series of nine patients with anti-IgLON5 disease
Source: Acta Neuropathol. 2023 Aug 30;146(4):631–45. doi: 10.1007/s00401-023-02625-6 (PMC10499680; doi:10.1007/s00401-023-02625-6)
Supplement: Supplementary file 1 — (DOCX 41 KB) [file 401_2023_2625_MOESM1_ESM.docx]

**Supplement**

**Supplementary Table 1.** Overview of lymphocytic infiltrates and granzyme B+ T cells in the brainstem of anti-IgLON5 disease

| Patient  ID, case reference | #1 *novel case* | #2 *reference no. [4]* | #3 *reference no. [5]* | #4 *reference no. [14]* | #5 *reference no. [24]* | #6 *reference no.* *[24]* | #7 *novel case* | #8 *reference no. [22]* | #9 *novel case* |
| --- | --- | --- | --- | --- | --- | --- | --- | --- | --- |
| Lymphocytic infiltrates in tegmentum of the brainstem | cells/mm^2^  perivascular  CD3+: 9  CD8+: 35  CD4+: 2  CD20+: 0  CD79a+: 2    parenchymal  CD3+: 41 CD8+: 37 CD4+: 9 CD20+: 0  CD79a+: 0 | cells/mm^2^  perivascular  CD3+: 7  CD8+: 9  CD4+: 0  CD20+: 4  CD79a+: 2  parenchymal  CD3+: 38 CD8+: 31 CD4+: 0  CD20+: 1 CD79a+: 0 | cells/mm^2^  perivascular  CD3+: 12  CD8+: 8  CD4+: 3  CD20+: 0  CD79a+: 18  parenchymal  CD3+: 42 CD8+: 40 CD4+: 3  CD20+: 1 CD79a+: 0 | cells/mm^2^ perivascular  CD3+: 70  CD8+: 63  CD4+: 33  CD20+: 5  CD79a+: 2  parenchymal  CD3+: 4 CD8+: 6 CD4+: 2 CD20+: 0 CD79a+: 0 | cells/mm^2^  perivascular  CD3+: 2  CD8+: 4  CD4+: 1  CD20+: n.a.  CD79a+: 7  parenchymal  CD3+: 4 CD8+: 5 CD4+: 0 CD20+: n.a.  CD79a+: 0 | cells/mm^2^  perivascular  CD3+: 11  CD8+: 10  CD4+: 2  CD20+: n.a.  CD79a+: 0  parenchymal  CD3+: 3 CD8+: 1 CD4+: 0 CD20+: n.a.  CD79a+: 0 | cells/mm^2^  perivascular  CD3+: 47  CD8+: 95  CD4+: 8  CD20+: 3  CD79a+: 9  parenchymal  CD3+: 5 CD8+: 5 CD4+: 4 CD20+: 0 CD79a+: 0 | n.a. | cells/mm^2^  perivascular  CD3+: 64  CD8+: 67  CD4+: 25  CD20+: 0  CD79a+: 0  parenchymal  CD3+: 15 CD8+: 22 CD4+: 7 CD20+: 3 CD79a+: 0 |
| Granzyme B + T cells/mm^2^  perivascular  parenchymal | 8 10 | n.a. | 3 5 | 1 3 | n.a. | n.a. | 4 3 | n.a. | 15 8 |

**Supplementary Table 2.** IgG1 and IgG4 deposits in depicted anatomic regions of anti-IgLON5 disease (case 2 not available)

| Patient ID |  | Hippocampus | Hypothalamus | Pons | Medulla oblongata | Cerebellum | Spinal cord |
| --- | --- | --- | --- | --- | --- | --- | --- |
|  |  | CA1 CA2 CA3 CA4 |  | Tegm. Base | Tegm. Ncl olivaris | Rinde. Ncl dentatus | AH PH Lam IV-VI |
| #1 | IgG1 | n.a. | n.a. | - - | - - | + n.a. | n.a. |
| *novel case* | IgG4 | n.a. | n.a. | - + | + + | + n.a. | n.a. |
| #3 | IgG1 | - - - - | - | - - | - - | + - | - - |
| *Ref. no. [5]* | IgG4 | - - - - | - | + ++ | +++ +++ | +++ +++ | - - |
| #4 | IgG1 | - - - - | - | - - | - - | + - | - - |
| *Ref.no. [14]* | IgG4 | - - - - | - | - - | - + | ++ - | - + |
| #5 | IgG1 | n.a. | n.a. | n.a. | n.a. | n.a. | n.a. |
| *Ref. no.[24]* | IgG4 | n.a. | - | n.a. | - - | + n.a. | n.a. |
| #6 | IgG1 | n.a. | n.a. | n.a. | n.a. | n.a. | n.a. |
| *Ref. no.[24]* | IgG4 | n.a. | - | n.a. | n.a. | - - | n.a. |
| #7 | IgG1 | - - - - | - | - - | n.a. | ++ - | n.a. |
| *novel case* | IgG4 | ++ ++ ++ - | ++ | + - | n.a. | +++ + | n.a. |
| #8 | IgG1 | - - - - | - | - - | - - | - - | - - |
| *Ref. no.[22]* | IgG4 | - - - - | + | ++ - | ++ - | + + | - - |
| #9  *novel case* | IgG1 | - - - - | - | - - | - - | - - | n.a. |
|  | IgG4 | - - - - | - | - - | + - | - - | n.a. |

**Video Supplement:** Patient 1 with PSP-phenotype eight years following symptom onset showing marked horizontal gaze-evoked nystagmus, postural instability on pull-test and broad-based, short-stepped gait with reduced arm swing.
